# Factors associated with eating performance in nursing home residents living with dementia and other comorbidities

**DOI:** 10.1186/s12877-024-05540-x

**Published:** 2024-11-15

**Authors:** Wen Liu, Kyuri Lee, Elizabeth Galik, Barbara Resnick

**Affiliations:** 1 University of Iowa College of Nursing, 50 Newton Rd., Iowa City, IA 52242 USA; 2 University of Maryland School of Nursing, 620 W Lombard St., Baltimore, MD 21201 USA

**Keywords:** Dementia, Cognitive impairment, Eating performance, Functional ability, Behavioral symptoms, Psychotropic medication use, Comorbidities, Nursing home

## Abstract

**Background:**

Eating performance is the functional ability to get food into the mouth and chew/swallow it. Nursing home residents with dementia commonly experience compromised eating performance and subsequent consequences. Prior work examined the association between resident eating performance and their cognitive and functional ability. Yet, the associations between resident eating performance and behavioral and psychological symptoms, psychotropic medication use, and comorbidities are less studied. This study aimed to examine the association between eating performance and cognition, functional ability, behavioral and psychological symptoms, psychotropic medication use, and comorbidities in nursing home residents with dementia.

**Methods:**

This was a secondary analysis using baseline data from two randomized controlled trials, testing the impact of Function Focused Care on function and behavioral symptoms in 882 residents with moderate-to-severe dementia (mean age 86.55 years, 71% female, 30% non-white, 68.5% severe dementia) from 67 nursing homes in two states between 2014 and 2020. Eating performance (dependent variable) was measured using the single self-feeding item of Barthel Index. Independent variables included cognitive impairment, functional ability (Barthel Index total score excluding the self-feeding item score), behavioral and psychological symptoms (agitation, depression, resistiveness-to-care), psychotropic medication use (anti-depression, sedative, anti-psychotics, anti-seizure, anti-anxiety), and comorbidities.

**Results:**

Nearly 39% of residents were dependent in eating. On average, residents had five documented comorbidities (SD = 3.06, range = 0–12) and were on approximately one psychotropic medication (SD = 1.25, range = 0–5). Eating performance was associated with cognitive impairment (OR = 0.53, 95% CI = 0.35, 0.79, *p* = .002), functional ability (OR = 1.05, 95% CI = 1.04, 1.06, *p* < .001), depressive symptoms (OR = 0.94, 95% CI = 0.89, 0.98, *p* = .007), and anxiolytic use (OR = 0.64, 95% CI = 0.42, 0.99, *p* = .046).

**Conclusions:**

Findings supported that better eating performance was associated with less cognitive impairment, higher functional ability, fewer depressive symptoms, and less anxiolytic use. Targeted interventions to accommodate to cognitive function, optimize functional ability, minimize anxiolytic use, and manage depressive symptoms are encouraged to support eating performance in residents with dementia.

## Background

Eating performance is defined as the functional ability to independently get food and fluids into the mouth and orally swallow them and is one of the most basic activities of daily living (ADLs) [1]. Maintaining eating performance is a critical indicator of physical and psychosocial quality of life, as it is not only essential for nutritional intake but offers opportunities to have positive mealtime and social experiences [2, 3]. Nearly one-third of residents living with dementia in nursing homes (NH) experience compromised eating performance [1, 4]. Compromised eating performance not only has a psychological and social impact on residents’ quality of life, but also leads to negative outcomes including weight loss, dehydration, malnutrition, and muscle weakness, and subsequently increased risks of morbidity and mortality [5–7].

Residents with dementia experience decline in functional and cognitive abilities and require different levels of assistance during mealtime. Further these residents may experience a decline or loss in their abilities to verbalize their preferences and needs and rely on nonverbal communication with care staff [8]. Inappropriate assessments of resident preferences and needs by staff based on limited verbal and mostly nonverbal communication with residents may result in inappropriate mealtime assistance such as directly feeding residents without adequately accommodating to their cognitive and functional abilities. Such mealtime assistance may lead to resident resistance to eat during mealtimes and may result in exacerbation of behavioral and psychological symptoms associated with dementia such as aggression, agitation, and resistiveness to care [9].

## Factors associated with eating performance

One way to comprehensively address the many factors that influence eating performance in residents with dementia is using the Social Ecological Model, which conceptualizes factors at intrapersonal (resident), interpersonal (staff), and environmental/institutional levels [10]. At the intrapersonal level, residents with dementia experience progressive changes in cognitive function (e.g., memory, language, orientation, problem-solving), biological and motor function (e.g., range of motion), sensory function (vision, taste, smell, hearing, touch), impaired oral health and hygiene (e.g., dental pain, dry mouth, denture problems), and physiological changes in the stages of chewing and swallowing (e.g., chewing ability, dysphagia, esophageal function) [3, 11, 12]. These changes are compounded by the high prevalence of comorbidities and use of medications, particularly psychotropics and potential side effects of sedation, leading to lack of alertness, changes in dietary habits, declines or loss of appetite, and impaired ability to see, hear, communicate, plan and perform complex mealtime activities, and tolerate the texture of regular food [1, 13–17]. All these factors reinforce less motivation and engagement in eating, less enjoyment of food, and low food intake [1, 13–17]. For example, several cross-sectional studies show that NH residents with severe cognitive impairment, compared with those with moderate impairment, are 2.7 times less likely to demonstrate independence in eating, and the likelihood of eating dependence increases with declining resident physical ability to engage in ADLs [1, 7]. Although not always consistently noted, other intrapersonal factors associated with eating performance include gender [18], duration of illness (dementia) [12], period of institutionalization [12], mood and behavioral symptoms [19, 20], grip strength [7], pain [20], and polypharmacy [17].

At the interpersonal level, the relationships and familiarity between direct care staff and residents as well as quality of mealtime care interactions are critical for resident eating performance, especially when residents require mealtime assistance, have chewing/swallowing difficulties, and/or eat slowly [3, 13, 21–24]. Staff provide most mealtime care and have the most opportunities to engage residents in eating. Yet, recent work shows that staff frequently miss opportunities to engage residents in eating, and often provide full assistance without evaluating and optimizing residents’ abilities and motivation to eat [3, 25]. For some residents, this care practice discourages their participation in eating and results in learned dependence where residents do not even attempt to eat independently and will just open their mouth when a utensil gets close; for other residents, this care practice may trigger resistive behaviors (e.g., turning their head away, refusing to open their mouth) during mealtime and result in disengagement and low food intake in residents [16, 26–28]. Conversely, providing verbal encouragement to eat independently and person-centered assistance tailored to resident needs and preferences increases resident engagement and food intake [25, 29, 30].

At the environmental/institutional-level, existing interventions targeting physical and social dining environments, routines, and institutional features demonstrate low-to-insufficient evidence on resident eating performance due to methodological limitations such as weak study designs and small samples [31–35]. However, recent work acknowledges the role of a supportive physical dining environment and institutional infrastructure (e.g., optimal lighting and sound levels, pleasant eating locations, sufficient staff support, adequate eating time) in fostering positive mealtime experiences [36–38]. Recent work also shows a high-quality dining environment that provides specific social stimuli including positive dyadic interactions tailored to resident needs and dietary preferences is associated with improved resident engagement in eating [25, 30, 39].

## Objectives

Prior work to date that examined intrapersonal factors associated with eating performance has mostly explored the role of cognitive and physical function. Limited research has focused on the role of specific behavioral and psychological symptoms, psychotropic drug use, or comorbid conditions. In addition, most of the prior studies are limited using small to medium sample sizes of residents with moderate-to-severe dementia.

The purpose of this study, therefore, was to examine the associations between resident eating performance and intrapersonal characteristics, including cognition, functional ability, behavioral and psychological symptoms (agitation, depression, resistiveness to care), psychotropic medication use (i.e., anti-depression, sedative, anti-psychotics, anti-seizure, anti-anxiety), and comorbidities. We hypothesized that residents with less cognitive impairment, better functional ability, fewer behavioral and psychological symptoms, fewer psychotropic medications, and fewer comorbidities would be more independent in eating, controlling for resident demographic characteristics (i.e., age, gender, race, marital status, education). Understanding the underlying intrapersonal factors associated with eating performance will guide the development and use of individualized, effective mealtime care strategies to improve eating performance in the growing aging population with dementia.

## Methods

### Design

This was a secondary analysis using baseline data from two longitudinal randomized controlled trials testing the impact of Function-Focused Care on function and behavioral symptoms among NH residents living with dementia between 2014 and 2020 in two East Coast states in the United States [40, 41].

### Sample and setting

In the parent trials, residents were eligible to participate if they were 55 years or older, were dwelling in participating NHs at the time of recruitment, had moderate or severe cognitive impairment with a Mini-Mental State Exam (MMSE) score of 15 or below [40] or a Brief Interview of Mental Status (BIMS) score of 12 or below [41], and were not receiving hospice care or subacute rehabilitation. Residents’ ability to self-consent was evaluated using the Evaluation to Sign Consent (ESC) [42]. If the resident failed the ESC test, they were asked to sign an assent form or verbally assent to participate, and proxy consent from the resident’s legally authorized representative was obtained. In total, 2,612 residents were screened based on inclusion criteria and 882 eligible residents from 67 long-term care (LTC) communities in two states consented to participate.

### Measures

Resident characteristics included age, gender, race, marital status, education, cognitive status, functional ability, behavioral symptoms (depressive symptoms, agitation, resistiveness to care), use of psychotropic medications, and comorbidities including dementia diagnosis. Data was collected by trained research assistants through abstraction of resident medical records (*demographics*,* medications*,* comorbidities)*, direct observation of residents *(resistiveness to care)*, resident interview* (cognition)*, and proxy reports from nursing staff *(functional ability*,* depressive symptoms*,* agitation)*.

**Cognitive status** was measured using different measures (MMSE and BIMS) in the two parent trials. MMSE is a screening tool that tests five domains of cognitive functions: orientation, registration, attention and calculation, recall, and language [43]. The scores range from 0 to 30, with higher score indicating less severe cognitive function; 19 ≤ MMSE ≤ 23 represents mild impairment, 10 ≤ MMSE ≤ 18 represents moderate impairment, and 0 ≤ MMSE ≤ 9 represents severe impairment [44]. BIMS is a cognition test that consists of 7 items of recall and orientation questions [45]. The scores range from 0 to 15, with higher score indicating better cognitive function; 8 ≤ BIMS ≤ 12 represents moderate impairment, and 0 ≤ BIMS ≤ 7 represents severe impairment. One of the eligibility criteria of the two parent trials was that residents had a MMSE score of 15 or below [40] or a BIMS score of 12 or below [41], indicating that all residents included in the two trials had moderate or severe cognitive impairment.

**Eating performance** was measured using the single self-feeding item from the modified Barthel Index (BI) [46]. This item is scored as follows: ‘0 = completely dependent and needs to be fed or partially dependent and much help needed’, ‘5 = limited help needed or partially independent and some help needed (such as with cutting food or spreading butter)’, to ‘10 = able to eat by self independently. In this study, eating performance (dependent variable) was skewed towards independent eating and, therefore, dichotomized as a binary variable: dependence in eating (self-feeding item score = 0 or 5) vs. independence in eating (score = 10).

**Functional ability** was evaluated using the modified Barthel Index [46], a 15-item functional assessment tool that measures an individual’s performance in ADLs. Each item is rated based on the degree of assistance needed from care staff. The total scores of Barthel Index, adding up 13 items and either the wheelchair 50 yards item or the walking 50 yards item, range from − 2 to 100. A maximum score of 100 indicates complete independence in self-care. The total score of 14 items, excluding the self-feeding item score, was used in this study with a possible range of -2 to 90.

**Depressive symptoms** were measured using the 19-item Cornell Scale for Depression in Dementia (CSDD) [47]. Each item is rated for severity with a 3-point Likert scale. Total scores range from 0 to 38, with higher score indicating more severe depressive symptoms.

**Agitation** was evaluated using the 14-item short form Cohen-Mansfield Agitation Inventory (CMAI) [48]. The scale assesses 14 agitated behaviors, with each being rated for frequency over the past two weeks with a 5-point scale using proxy-report. Total scores range from 14 to 70, with higher score indicating more agitation.

**Resistiveness to care** was measured by the 13-item Resistiveness to Care Scale. This scale is an observational tool that assesses 13 resistive behaviors (clench mouth, turn away, push/pull, push away, pull away, grab person, grab object, scream/yell, cry, threaten, hit/kick, say no, adduct) that commonly occur in individuals with dementia during care interactions [49]. The score of each behavior (0 = not observed and 1 = observed) adds up to the total score ranging from 0 to 13. The total scores of the study sample were highly skewed toward less resistiveness to care and, therefore, resistiveness to care was dichotomized as a binary variable (0 = none of the 13 resistive behaviors was observed, 1 = at least one of the 13 resistive behavior was observed).

**The use of any medication from five categories of psychotropic medications** was considered as evidence of psychotropic medication use and included: antidepressants, sedatives, antipsychotics, anticonvulsants, and anxiolytics. Evidence was based on chart review of all regular administration of these medications. In addition to individual use of a drug group, a total number of psychotropic medications prescribed and used regularly was calculated to represent polypharmacy status. Both total and individual psychotropic medication use were used to describe sample characteristics and only individual psychotropic medication use was included in the regression model due to multicollinearity.

**Comorbidities** was measured by extracting data from medical records and calculated using the Cumulative Illness Rating Scale to sum the 14 illnesses included in the measure [50]. 

### Data analysis

All analyses were performed with SPSS 25.0 (IBM corp, Armonk, NY, USA). A two-sided α < 0.05 was considered significant in all analyses. Descriptive statistics were used to present sample characteristics. Chi-squared and t-tests were performed to compare categorical and continuous independent variables (i.e., cognitive impairment, functional ability, behavioral and psychological symptoms, number of comorbidities, use of individual types of psychotropic medication) and resident characteristics (i.e., age, gender, marital status, race, education), respectively, between residents with independence vs. dependence in eating.

Binary logistic regression model was fit to examine the associations between independent variables of interest (resident cognitive impairment, functional ability, behavioral and psychological symptoms, use of individual types of psychotropic medication, and number of comorbidities) and eating performance, controlling for resident demographic characteristics (i.e., age, gender, race, marital status, education). Adjusted Odds ratio (OR), 95% confidence interval (95% CI), and coefficient (β) of parameters were computed. The extent of multicollinearity among independent variables of interest were examined using the VIF values: (1) the largest VIF values less than 1 indicating no concern, between 1 and 5 indicating mild concern, between 5 and 10 indicating moderate concern, and more than 10 indicating a serious problem; and (2) the average VIF values substantially greater than 1 indicating the regression may be biased [51]. Omnibus test and Hosmer and Lemeshow test were used to assess the goodness of fit for the model: significant Omnibus test and non-significant Hosmer and Lemeshow test indicate that the model is significant. Nagelkerke pseudo R^2^ statistic for the model was calculated to measure the percent of variance explained by independent variables of interest in eating performance.

The patten and mechanism of missing data were examined. All the variables had none or very small missing (0.7% for comorbidities, 1.1% for depressive symptoms, 1.2% for age, gender, race, and medications, 1.4% for resistiveness to care, 1.6% for marital status and agitation, 02.2% for education and functional ability, 1.9% for cognitive impairment), which did not exceed the 5–10% recommended allowable limit for missing [52]. Data were considered as missing completely at random (MCAR) based on the findings that there were no statistically significant differences between any pair of variables using the separate variance t tests and that the Little’s MCAR test was not significant (chi-square = 21.93, *p* = .188) using list wise deletion with expectation maximization [52]. All missing data were treated as missing systematically and were not imputed for all analyses.

## Results

### Sample characteristics

Nearly 39% of residents (*n* = 339) were dependent in eating. Among the 339 residents, 16% were partially dependent and needed limited to some help from staff (Barthel Index self-feeding item score = 5) and 23% were completely dependent and needed extensive to full assistance from staff (Barthel Index self-feeding item score = 0) during mealtime.

Resident characteristics are shown in Tables 1 and 2. Residents’ mean age was 87 years old (SD = 10.34), ranging from 58 to 107. The majority were female (71.3%), white (68.5%; African American 30%, Asian or more than one race 0.3%), and unmarried (i.e., those who were separated, widowed, divorced, or never married; 71.2%). Resident education level was primarily elementary (39.3%) or unknown (35.6%). Most residents had severe dementia (68.5%) and did not exhibit resistiveness to care (81.4%). Residents had a moderate level of functional ability with a mean BI score of 46.4 excluding the item related to eating (SD = 30.68), had low levels of depressive symptoms and agitation with a mean CSDD score of 4.25 (SD = 4.35) and CMAI score of 20.63 (SD = 4.35). On average, residents had five documented comorbidities (SD = 3.06) and were on one psychotropic medication (SD = 1.25). The most common psychotropics used were antidepressants (35.7%), followed by antipsychotics (20.6%), anxiolytics (19.5%), and anticonvulsants (19.3%); sedatives were rarely used (0.5%). Bivariate analyses (Table 3) showed that resident eating performance was associated with education, cognitive status, functional ability, depressive symptoms, agitation, resistiveness to care, comorbidities, and use of anxiolytics. Resident age, gender, marital status, race, and the use of antidepressants, sedatives, antipsychotics, and anticonvulsants were not different by eating performance.

**Table 1:** Resident characteristics – continuous variables

Variables (measures)	Range	Mean ± SD
Age (years), (*n* = 871)	58–107	86.55 ± 10.34
Functional ability (BI)^a^ (*n* = 863)	-2-105	31.67 ± 30.68
Depressive symptoms (CSDD) (*n* = 872)	0–28	4.25 ± 4.35
Agitation (CMAI) (*n* = 868)	14–54	20.63 ± 7.66
Comorbidities (number of diseases) (*n* = 876)	0–12	5.36 ± 3.06
Polypharmacy (number of psychotropic medications) (*n* = 871)	0–5	1.16 ± 1.25

BI, Barthel Index; CSDD, Cornell Scale for Depression in Dementia; CMAI, Cohen-Mansfield Agitation Inventory. ^a^ The self-feeding item was excluded

**Table 2:** Resident characteristics – categorical variables

Variables	Categories		*n* (%)
Gender (*n* = 871)	Male		242 (27.4)
	Female		629 (71.3)
Marital status (*n* = 868)	Never		136 (15.4)
	Married		188 (21.3)
	Widowed/Divorced/Separated		492 (55.8)
	Do not know		52 (5.9)
Education (*n* = 863)	Elementary school (1–8 years)		347 (39.3)
	High school (9–12 years)		125 (14.2)
	College/Post-college		77 (8.7)
	Do not know		314 (35.6)
Race (*n* = 871)	White		604 (68.5)
	African American/Asian/More than one race		267 (30.3)
Cognitive impairment (*n* = 865)	Moderate		261 (29.6)
	Severe		604 (68.5)
RTC (*n* = 870)	Yes		152 (17.2)
	No		718 (81.4)
Medication use (*n* = 871)			
Antidepressants	Yes		315 (35.7)
	No		556 (63.0)
Sedatives	Yes		4 (0.5)
	No		867 (98.3)
Antipsychotics	Yes		182 (20.6)
	No		689 (78.1)
Anticonvulsants	Yes		170 (19.3)
	No		701 (79.5)
Anxiolytics	Yes		172 (19.5)
	No		699 (79.3)

RTC, Resistiveness to Care

**Table 3:** Comparison of resident characteristics by eating performance

Continuous Variables (measures)			Dependent (*n* = 339)	Independent (*n* = 532)	t	*p* value
			Mean ± SD			
Age (yrs), (*n* = 871)			87.22 ± 10.35	86.10 ± 10.35	1.57	0.118
Functional ability (BI)^a^ (*n* = 863)			13.72 ± 18.41	43.08 ± 31.47	-17.27	< 0.001*
Depressive symptoms (CSDD) (*n* = 872)			4.90 ± 4.59	3.84 ± 4.16	3.51	< 0.001*
Agitation (CMAI) (*n* = 868)			21.36 ± 8.28	20.18 ± 7.22	2.13	0.033*
Comorbidities (number of diseases) (*n* = 876)			4.79 ± 3.21	5.71 ± 2.91	-4.26	< 0.001*
**Categorical Variables**			**N (%)**		**χ** ^**2**^	***p*** **value**
Gender (*n* = 871)	Male		91 (26.8)	151 (28.4)	0.25	0.621
	Female		248 (73.2)	381 (71.6)		
Marital status (*n* = 868)	Never		46 (13.6)	90 (16.9)	2.88	0.410
	Married		81 (24.0)	107 (20.2)		
	Widowed/Divorced/Separated		190 (56.4)	302 (56.9)		
	Do not know		20 (5.9)	32 (6.0)		
Education (*n* = 863)	Elementary school (1–8 years)		169 (50.3)	178 (33.8)	25.96	< 0.001*
	High school (9–12 years)		37 (11.0)	88 (16.7)		
	College/Post-college		20 (6.0)	57 (10.8)		
	Do not know		110 (32.7)	204 (38.7)		
Race (*n* = 871)	White		234 (69.0)	370 (69.5)	0.03	0.871
	African American/Asian/More than one race		105 (31.0)	162 (30.5)		
Cognitive impairment (*n* = 865)	Moderate		78 (23.3)	183 (34.5)	12.32	< 0.001*
	Severe		257 (76.7)	347 (65.5)		
RTC (*n* = 870)	Yes		78 (23.0)	74 (13.9)	11.81	0.001*
	No		261 (77.0)	457 (86.1)		
Medication use (*n* = 871)						
Antidepressants	Yes		132 (38.9)	183 (34.4)	1.85	0.174
	No		207 (61.1)	349 (65.6)		
Sedatives	Yes		2 (0.6)	2 (0.4)	0.21	0.649
	No		337 (99.4)	530 (99.6)		
Antipsychotics	Yes		68 (20.1)	114 (21.4)	0.24	0.628
	No		271 (79.9)	418 (78.6)		
Anticonvulsants	Yes		77 (22.7)	93 (17.5)	3.61	0.057
	No		262 (77.3)	439 (82.5)		
Anxiolytics	Yes		81 (23.9)	91 (17.1)	6.02	0.014*
	No		258 (76.1)	441 (82.9)		

* *p <* .05. BI, Barthel Index; CSDD, Cornell Scale for Depression in Dementia; CMAI, Cohen-Mansfield Agitation Inventory, RTC, Resistiveness-to-Care. ^a^ The self-feeding item was excluded

### Factors associated with eating performance

Based on the binary logistic regression model (Table 4), less cognitive impairment (OR = 0.53, 95% CI = 0.35, 0.79, *p* = .002), better functional ability (OR = 1.05, 95% CI = 1.04, 1.06, *p* < .001), fewer depressive symptoms (OR = 0.93, 95% CI = 0.89, 0.98, *p* = .004), and less anxiolytics use (OR = 0.63, 95% CI = 0.41, 0.98, *p* = .039) were significantly associated with better eating performance. Resident agitation, resistiveness-to-care, and number of comorbidities were not significantly associated with eating performance. Regarding covariates, resident eating performance was significantly associated with their education level, and not associated with their age, gender, race, and marital status. The VIF values for all the independent variables (mean = 1.213, range = 1.046–1.315) were close to 1, indicating that multicollinearity was not a concern. The model was significant (Omnibus test *p* < .001; Hosmer and Lemeshow test *p* = .624), classifying 75% of the residents and explaining 40.5% of the variance in eating performance (Negelkerke R^2^ = 0.405).

**Table 4:** Binary logistic regression model to predict eating performance (*N* = 845)

Variables (measures)	β	OR	95% CI	*p* value
Age	-0.001	1.00	(1.98, 1.02)	0.890
Gender (0 = male, 1 = female)	-0.071	0.93	(0.62, 1.41)	0.737
Marital status				
Never (reference)				
Married	-0.116	0.89	(0.50, 1.58)	0.690
Widowed/Divorced/Separated	0.072	1.07	(0.64, 1.82)	0.789
Don’t know	0.532	1.70	(0.75, 3.85)	0.201
Race (0 = White, 1 = African American/Asian/More than one race)	0.015	1.02	(0.68, 1.51)	0.942
Education				
Elementary school (reference)				
High school	0.546	1.73	(0.84, 3.53)	0.135
College/Post-college	1.111	3.04*	(1.33, 6.91)	0.008
Don’t know	0.739	2.09*	(1.27, 3.45)	0.004
Cognitive impairment (0 = moderate, 1 = severe)	-0.636	0.53*	(0.35, 0.79)	0.002
RTC (0 = no, 1 = yes)	0.178	1.20	(0.76, 1.87)	0.438
Functional ability (BI)	0.050	1.05*	(1.04, 1.06)	< 0.001
Depression (CSDD)	-0.071	0.93*	(0.89, 0.98)	0.004
Agitation (CMAI)	-0.015	0.99	(0.96, 1.01)	0.297
Comorbidities	0.066	1.07	(0.99, 1.16)	0.106
Antidepressants (0 = no,1 = yes)	-0.050	0.95	(0.65, 1.39)	0.795
Sedatives (0 = no,1 = yes)	-1.418	0.24	(0.02, 3.43)	0.294
Antipsychotics (0 = no,1 = yes)	0.112	1.12	(0.73, 1.72)	0.607
Antiseizure medications (0 = no,1 = yes)	-0.305	0.74	(0.47, 1.16)	0.187
Antianxiety medications (0 = no,1 = yes)	-0.459	0.63*	(0.41, 0.98)	0.039

* *p <* .05. OR = Odds Ratio. CI = Confidence Interval. RTC, Resistiveness to Care; BI, Barthel Index; CSDD, Cornell Scale for Depression in Dementia; CMAI, Cohen-Mansfield Agitation Inventory. Omnibus Tests: *P* < .001; Hosmer and Lemeshow Tests: *P =* .624. Negelkerke R^2^ = 40.5%

## Discussion

This study examined associations between eating performance and intrapersonal characteristics in NH residents with moderate to severe dementia. Our findings partially supported the hypothesized associations: four intrapersonal variables – cognitive impairment, functional ability to perform ADLs, depressive symptoms, and use of anxiolytic medication – were identified as significant factors associated with eating performance. Particularly, cognitive impairment and anxiolytic medication use were most strongly associated with eating performance, followed by depressive symptoms and functional ability.

While the study sample had moderate to severe dementia, our findings support better eating performance was associated with lower cognitive impairment, consistent with prior studies [1, 7, 39]. Thereby, staff assistance needs to attend to residents’ cognitive function during mealtime care, such as orientation to the time and place of meals, verbal and nonverbal communication and expressions of needs and preferences, naming and labeling meal-related items such as food and drinks. Optimizing and accommodating to remaining cognitive and functional abilities in residents with moderate to severe cognitive impairment is particularly important to actively engage residents in mealtime activities. Common strategies that are likely helpful include providing appropriate types of food and drinks that accommodate to the resident’s remaining cognitive abilities, simplifying the presentations of food and drinks, and limiting stimulation from the physical and social dining environments [25]. For example, staff can present single food or drink items at a time rather than a whole tray of food and drink items, provide finger foods that residents can eat with their hands instead of food options that require utensils, and minimize table clutter to decrease stimulation and the risk of being distracted between food and other options to help engage individuals with moderate to severe dementia to perform eating activities [53].

Of the five types of psychotropics, anxiolytic medication use was the only significant factor: less use of anxiolytics was associated with better eating performance. Consistent with our findings, a one-year prospective cohort study on nursing home residents with middle-stage dementia showed no significant associations between eating disability and antipsychotics and antidepressants [4]. This study also noted no association between eating disability and use of benzodiazepines (one type of anxiolytics and sedatives) [4]. Conversely, a cross-sectional study in acute psychogeriatric inpatient care settings showed better ADL function (including eating function) was associated with less daily dose of benzodiazepines rather than other psychotropic medications [54]. The findings in our study may have been different because we focused on the association of eating performance (i.e., actual functional performance during mealtime) with the use of all anxiolytic medications and the use of all sedatives medications, rather than the association between the use of benzodiazepines alone and eating disability (i.e., lack of ability to eat independently). The potential side effects of anxiolytics, such as drowsiness, dizziness, and worsening cognition may hinder independence in performing ADLs including eating [55]. Our findings suggest the need to minimize inappropriate use of anxiolytics for optimal eating performance in residents with dementia. Person-centered, nonpharmacological approaches are recommended as first-line management given risks associated with the use of these medications.

Depressive symptoms were found to be another significant intrapersonal factor: the higher the level of depressive symptoms, the more likely the resident was dependent in eating. While prior studies with much smaller samples did not identify any significant association between eating performance and depressive symptoms [1, 7], some reports indicated that higher level of depressive symptoms were associated with an increased risk of ADL limitations [56, 57]. It is reasonable that residents who experience more depressive symptoms are less likely to actively engage in eating activities, and care staff may keep engaging residents and provide essential assistance to assure that they have sufficient food intake. Our findings suggest care staff need to be aware of the link between depressive symptoms and decline in eating performance and use innovative approaches to engage individuals in independent eating. Some care strategies provided by staff may include making the mealtime experience fun with stimulating physical and social dining environments including pleasant and preferred music, eating with the resident if allowable, and offering preferred food options.

As previously noted [1, 7], our findings indicated that agitation and resistiveness to care were not associated with eating performance. The lack of significance may be due to the low frequency of behavioral symptoms in our sample. Resistiveness to care was observed in only 17% of the residents. It is possible that agitated and resistive behaviors were masked by psychotropic and other medications that are frequently used in LTC settings to control challenging behavioral and psychological symptoms [58]. In addition, resident agitation and resistiveness to care were measured during varied care interactions in addition to during mealtimes (e.g., during bathing or dressing) in this study. Future research should consider noting specifically during which ADL care interaction each behavioral symptom occurred.

Findings show that functional ability to perform ADLs was positively associated with eating performance such that improvement in overall ADL performance may improve eating performance. This finding is consistent with previous reports that supported the association between eating performance and functional ability [1, 12, 17]. Eating requires upper extremity motor skills, muscle strength, and hand-eye coordination to use utensils, bring food to the mouth, hold and chew food in the mouth, and swallow food. Therefore, assessing and supporting functional ability through tailored interventions can promote independence in eating activities. Existing studies have revealed that physical exercise or occupational therapy could help enhance strength, mobility, balance, and visuospatial functioning in residents with dementia and thereby improve eating performance [59–61]. In addition, the use of finger foods or assistive devices (e.g., cutlery with adjusted angles and thicker handles, modified cups and plates, non-slip mats) may optimize remaining functional abilities and facilitate eating performance among residents with dementia and decreased physical abilities [62, 63]. These strategies can be especially helpful for those with comorbid Parkingson’s disease or stroke who experience unintended or uncontrollable movements or muscle weakness and have difficulties with balance and coordination of arms, hands, and/or fingers.

The association of comorbidities with eating performance was not supported in this study. This finding is consistent with a prior study [1], and inconsistent with other studies which found significant associations [4, 7]. The discrepancy between studies may be partly explained by different sample sizes, dementia severity of study samples, measurement approaches, geographical regions, as well as how comorbidities were accessed (e.g., survey/interview vs. medical record review). For example, while some studies used the number of comorbidities [1, 7], another study measured comorbidities using Charlson’s Comorbidity Index [64] that assigns weighted scores to various comorbid conditions based on estimated relative risk of mortality [4]. Assessments that consider not only the number, but also the type, severity, and intensity of different comorbid conditions may be useful in future studies. For example, different types of comorbidities may have different impacts on eating performance: hypertension may not have any direct impact, arthritis, neurological and respiratory conditions are more likely to have some impact, whereas conditions related to eyes, ears, nose, oral/teeth, throat, larynx, and/or upper gastrointestinal tract very likely have some impact on eating performance. Future research may control for specific comorbid conditions that likely have impact on eating performance rather than all comorbidities.

## Implications for clinical practice

The Centers for Medicare & Medicaid Services, HHS’ long-standing regulations for high quality mealtime care in tong-term care settings include, but not limited to, offering sufficient drinks to promote hydration, offering at least three meals daily at regular and normal mealtimes, offering assistive devices and modified diets as needed, and having trained staff provide mealtime assistance with supervision. These regulations delineate the updated Minimum Staffing Standards (including RN staffing) for long-term care during mealtimes and warrant the urgent needs to improve LTC staffing to reduce the risk of residents receiving unsafe and low-quality care [65]. Consistently with the regulations, findings of this study indicate the need to attend to resident cognitive status, functional ability, depressive symptoms as well as use of anxiolytics before, during, and after mealtimes, when nursing home staff assist residents to eat. To ensure safe and high-quality care and maintain resident eating performance as much as possible, adequate and high-quality staffing is needed for mealtime care of nursing home residents with dementia.

## Strengths and limitations

The strengths of this study are the use of a large, diverse sample by gender and race from a large number of LTC communities, as well as the use of a variety of resident characteristics, including function, behaviors, medication use, and comorbidities as independent variables. The study has several limitations. While the study sample is diverse with more than 30% being non-white, the racial differences on likes, dislikes, restrictions, and culture related to food and diet was not considered in the analysis. Eating performance was measured using one self-care feeding item from the BI scale, which provides clear cutoffs for grouping residents but may not reflect the whole spectrum of functional ability required for eating during mealtime. The use of a single-item measure to assess the dependent variable may lead to potential measurement bias. Additionally, it was not specified whether the observed resident eating dependence and the need for mealtime assistance were due to a lack of willingness or disability to eat in residents. Interaction effects among individual independent variables of interests were not included in the model because this study focused on the association of individual independent variables of interest and the dependent variable rather than the interaction effects, and adding interaction terms will tremendously increase model complexity and could lead to model overfitting, making it harder to interpret the individual effects of the variables. Two different measures of cognitive function (MMSE and BIMS) were used across the two parent studies. The study sample showed low levels of behavioral symptoms, as individuals with higher levels of behavioral symptoms tend to be less likely to assent to research. Furthermore, resistiveness to care was measured by observing resident responses in various ADLs (rather than exclusively during mealtime) for a limited period (rather than the whole duration) of care interactions during ADLs, and therefore, may have missed some of the resistive behaviors. The study only included residents with moderate to severe cognitive impairment residing in LTC communities from two states in the Eastern region, and findings may not generalize to residents with mild or no impairment.

## Directions for future research

While this study examined the role of multiple intrapersonal factors using a large, racially diverse sample, three directions for future research are informed. First, future studies may benefit from using psychometrically validated multi-item measures that assess various aspects of eating performance, such as the Level of Eating Independence Scale [66] and the Eating Behavior Scale [67]. Second, prior studies show that resident behavioral symptoms including resistive behaviors (73.6–78.2%) are more frequently observed during mealtime compared to other ADLs (4.3–6.4%), and resident positive behaviors are also more commonly observed during mealtime (50.9–68.2%) compared to other ADLs (72-92.4%) [16, 27, 68–72]. Therefore, further research is necessary to examine the role of resident behavioral symptoms as well as positive behaviors, specifically within the context of mealtime care interactions, on eating performance. Third, future research may examine factors at the staff-, dyadic- and environmental/institutional- levels, such as the quality of staff mealtime engagement using Mealtime Engagement Scale [73, 74], the quality of dyadic care interactions using the Quality of Care Interaction Survey [75, 76], as well as physical, social, and cultural dining environments, and institutional cultures/policies associated with mealtime [3].

## Conclusion

This study identified multiple intrapersonal factors that were associated with eating performance among LTC residents with moderate-to-severe cognitive impairment. Lower cognitive impairment, better functional ability, lower levels of depressive symptoms, and less anxiolytic medication use were predictive factors of eating independence. Findings suggest a need to prepare care staff for multifactorial assessments of these intrapersonal factors as well as to provide tailored care to preserve independence in ADL function, minimize the use of anxiolytic drugs, and manage depressive symptoms to optimize eating performance in residents with dementia. Findings also provide directions for future dementia mealtime care research and guide the development and use of individualized, resident-centered mealtime care strategies to improve and maintain mealtime independence.

## Data Availability

The datasets used and/or analyzed during the current study are available from the corresponding author on reasonable request.

## Abbreviations

ADLs: Activities of daily living
BI: Barthel Index
BIMS: Brief Interview of Mental Status
CI: Confidence interval
CMAI: Cohen-Mansfield Agitation Inventory
CSDD: Cornell Scale for Depression in Dementia
LTC: Long-term care
MMSE: Mini-Mental State Exam
NH: Nursing home
OR: Odds ratio
RTC: Resistiveness to care
